# *Moraxella catarrhalis* Activates Murine Macrophages through Multiple Toll Like Receptors and Has Reduced Clearance in Lungs from TLR4 Mutant Mice

**DOI:** 10.1371/journal.pone.0037610

**Published:** 2012-05-25

**Authors:** Ferdaus Hassan, Dabin Ren, Wenhong Zhang, Tod J. Merkel, Xin-Xing Gu

**Affiliations:** 1 Vaccine Research Section, National Institute on Deafness and Other Communication Disorders, Rockville, Maryland, United States of America; 2 Center for Biologics Evaluation and Research, Food and Drug Administration, Bethesda, Maryland, United States of America; Charité-University Medicine Berlin, Germany

## Abstract

*Moraxella catarrhalis* is a Gram negative bacterium and a leading causative agent of otitis media (OM) in children. Several recent reports have provided strong evidence for an association between toll like receptors and OM. It has been found that both *Streptococcus pneumoniae* and nontypeable *Haemophilus influenzae* activate host protective immune responses through toll like receptors (TLRs), however, the precise mechanism by which *Moraxella catarrhalis* initiates the host immune response is currently unknown. In this report, using murine macrophages generated from a series of knock-out mice, we have demonstrated that *M. catarrhalis* lipooligosaccharide (LOS) and either heat killed or live bacteria are recognized by one or more TLRs. LOS activates the host immune response through a membrane bound CD14-TLR4 complex, while both heat killed and live *M.cat* require recognition by multiple toll like receptors such as TLR2, TLR4 and TLR9 without the requirement of CD14. We have also shown that *M.cat* stimuli are capable of triggering the host innate immune response by both MyD88- and TRIF- dependent signaling pathways. We further showed that *M.cat* induced activation of mitogen activated protein kinase (MAPK) is essential in order to achieve optimal secretion of pro-inflammatory cytokine TNF-α. We finally showed that TLR4 mutant C3H/HeJ mice produce significantly lower levels of pro-inflammatory cytokines TNF-α and IL-6 *in vivo*, An increased bacterial loads at 12 and 24 hours (*P<0.001*) in their lungs upon challenge with live *M.cat* in an aerosol chamber compared to wild-type (WT) control mice. These data suggest that TLRs are crucial for an effective innate immune response induced by *M.cat*. The results of these studies contribute to an increased understanding of molecular mechanism and possible novel treatment strategies for diseases caused by *M.cat* by specifically targeting TLRs and their signaling pathways.

## Introduction

The innate immune system is critical for the initiation of effective immune response against invading pathogens. Toll like receptors (TLRs) are pattern recognition receptors that can ‘sense’ a wide range of microbial products, provoke host responses and produce various pro- and anti-inflammatory cytokines such as TNF-α, IL-6, IL-12, IFN-γ, NO, IL-10 etc. that trigger both innate and adaptive immune responses [1]. So far, 10 TLRs in human and 12 TLRs in mice have been identified. Lipopeptides and other components of Gram positive bacteria are recognized by TLR2 in conjunction with either TLR1 or TLR6, lipopolysacharride (LPS) is recognized by TLR4, flagellin is detected by TLR5, unmethylated DNA and CpG-oligodeoxynucleotides (CpG-DNA) by TLR9, double-stranded RNA (dsRNA) or poly I:C by TLR3 and single stranded RNA (ssRNA) by TLR7 and TLR8 [2]. Most of the receptors that detect bacterial products are located on cells surface (TLR2, TLR4, TLR5) while other receptors that can sense nucleic acid are located in intracellular compartments such as endosome (TLR3, TLR7, TLR8, TLR9) [3]. Once TLRs recognize microbial products, they activate distinct signaling pathways through different adaptor molecules such as MyD88, Mal, TRIF and TRAM, leading to the activation of various transcription factors (NF-κB, IRFs, AP-1 etc.) [4]. Among all the TLRs, only TLR4 has the unique ability to activate both MyD88-dependent and the TRIF-dependent signaling pathways, the later initiates the type-1 interferon response as well as late NF-κB activation [5], [6].

Several clinical studies have shown the association between TLRs and otitis media (OM). Lee *et al.* recently reported that TLR2 and TLR4 are expressed in all the middle ear fluid samples of OM with effusion, but found no correlation between the expression of TLRs and concentration of immunoglobulin [7]. The same group also reported a significantly lower expression of TLR9 in otitis-prone group than in non-otitis-prone group [8]. Gene expressions of TLR3, TLR4, TLR5 and TLR7 were significantly lower in patients with chronic middle ear disease compared to control patients [9]. On the other hand, increased expressions of TLRs also have been reported. Granath *et al.* found increased TLR7 expression in the adenoids of children with OM with effusion [10]. Another study by Szczepanski *et al*. reported a distinct expression of TLR2, TLR3, and TLR4 in tissues isolated from human acquired cholesteatoma [11].

Until recently, *Streptococcus pneumoniae* was recognized as the leading causative pathogen of OM; however, due to the introduction of newly developed heptavalent pneumococcal vaccine, the relative contribution of *S. pneumoniae* to OM has changed and nontypeable *Haemophilus influenzae* (NTHi) has become the leading cause of infection, and *Moraxella catarrhalis (M.cat)* now ranks third. Mounting evidence suggests an active and critical role of TLRs in OM. It has been reported that TLR2 and p38 MAPK are important for NTHi induced NF-κB activation in human epithelial cells [12]. *In vivo* studies with NTHi showed distinct involvement of TLRs in different settings of animal models. Van Der Poll and his colleagues found that MyD88-dependent TLR4 signaling is important in clearing NTHi from the mouse lung [13]. On the other hand, Ryan and colleagues showed that deletion of TLR9 significantly prolonged the inflammatory response induced by NTHi and delayed bacterial clearance [14]. In a pneumococcal disease model, TLR2^−/−^ mice showed reduced clearance during later stages of pneumococcal colonization. When *S. pneumoniae* were inoculated in the middle ear of TLR2^−/−^ mice, almost 50% of the mice died within 3 days of inoculation compared to WT control mice. Of those TLR2^−/−^ mice that survived, more severe hearing loss was observed [15]. Similar to TLR2, TLR4^−/−^ and TLR9^−/−^ mice also showed higher bacterial loads and reduced survival against pneumococcal infection [16], [17].

*Moraxella catarrhalis* is an exclusive human pathogen and one of the leading infectious agent that causes OM in children and also found in patients with chronic obstructive pulmonary diseases (COPD) [18], [19]. *M.cat* is responsible for 10%–20% of all episodes of OM in infants and children [20], [21]. However, compared to NTHi and *S. pneumoniae*, our knowledge about *M.cat*-induced host immune response is lacking. Previously we reported that the lipooligosaccharide (LOS), a key virulence factor of *M.cat* activates human monocytes through CD14-TLR4 [22]. Schaar *et al*. recently reported that outer membrane vesicles (OMV) of *M.cat* initiate host responses through TLR2 [23]. *M.cat* also has the ability to invade bronchial epithelial cells and primary airway epithelial cells and initiates a TLR2 and partly NOD1-dependent inflammatory response [24]. However, the details of the signaling pathways that are activated by either *M.cat* LOS or whole bacteria, to our knowledge, is not completely known. In the present study, we found that LOS from *M. cat* is detected by murine macrophages exclusively through CD14-TLR4 complex, while other TLRs including TLR2 and TLR9, in addition of TLR4, detect either heat-killed (HK) or live bacteria and induce pro-inflammatory cytokines through both MyD88-dependent and TRIF-dependent signaling pathways. Finally, we showed that in a model of *in vivo* aerosol challenge of mice, the TLR4 mutant C3H/HeJ strain produces significantly lower amounts of TNF-α and IL-6 compared to WT C3H/OuJ mice and bacterial clearance was significantly delayed at late time points in mutant mice. Together, we have shown that TLR4 plays an important protective role against *M.cat* infection.

## Materials and Methods

### Mice

Female mice, 6–8 wks old, were used for all experiments. CD14^−/−^ (strain name B6.129S-*Cd14^tm1frm^*/J), TLR2^−/−^ (strain name B6.129-*Tlr2^tm1Kir^*/J), TRIF^−/−^ (strain name C57BL/6J-*Ticam1^Lps2^*/J), WT control C57BL/6J, LPS-unresponsive TLR4 mutant C3H/HeJ, and WT control C3H/OuJ mice, were purchased from Jackson Laboratory, Bar Harbor, ME. TLR4^−/−^and MyD88^−/−^ mice were originally described by Shizuo Akira (University of Osaka, Japan) and were bred at the University of Maryland, School of Medicine (Baltimore, MD, USA), and kindly provided by Dr. Stefanie N. Vogel. These mice were backcrossed onto a C57BL/6 background for ≥8 generations prior to use and genotyped to ensure their genetic status. TLR2^−/−^/TLR4^−/−^/TLR9^−/−^ triple knock-out mice were generated by first crossing TLR2 and TLR9 KO mice to generate TLR2/9 KO mice. The presence of wild-type vs. TLR2 or TLR9 KO alleles was determined for each offspring by PCR analysis of chromosomal DNA isolated from whole blood. Once identified, the TLR2/9 KO mice were crossed with TLR2/4 KO mice to generate TLR2/4/9 KO mice. The presence of wild-type vs. TLR2, TLR4 or TLR9 KO alleles was determined for each offspring by PCR analysis of chromosomal DNA isolated from whole blood. All experiments involving mice were performed according to the recommendations in the Guide for the Care and Use of Laboratory Animals of the National Institutes of Health. Protocol was reviewed and approved by institutional review board at the National Institutes of Health (Permit Number: 1158).

### Bacterial strain and purified LOS

*M. catarrhalis* strain 25238 (serotype A) was purchased from the American Type Culture Collection (Manassas, VA). LOS purification from type A strain has been described earlier [25]. The protein and nucleic acid in purified LOS was less than 1%.

### Reagents

Lipopolysaccharide (LPS) from *E.coli* 055:B5 was purchased from Sigma Chemicals (St Louis, MO). The JNK1/2 inhibitor (SP600125), p38 inhibitor (SB202190), and ERK1/2 inhibitor (U0126) were obtained from Calbiochem (San Diego, CA). and recombinant mouse M-CSF was purchased from R&D systems (Minneapolis, MN). The TLR2 ligand ‘synthetic triacylated lipoprotein’ (Pam3CSK4), the TLR3 ligand (poly I:C), and the TLR9 ligand CpG DNA were purchased from InvivoGen (San-Diego, CA).

### Preparation of bone marrow-derived macrophage

Bone marrow-derived macrophages (BMMØ) were prepared as described earlier [26], [27] with modification. Mouse tibias and femurs were cut at both ends and flushed with RPMI 1640 medium containing 10% FBS. Harvested bone marrow cells were cultured in the presence of murine recombinant M-CSF at 20 ng/ml for 7 days at 37°C under 5% CO_2_. Every two days, media was replaced with new media containing M-CSF at same concentration. At day 7, adherent macrophages were used for further experiment (in the absence of M-CSF). Flow cytometric analysis showed that >90% cells were CD11b-, F4/80- and MHC II-positive.

### Cell stimulation

BMMØ were stimulated with LOS (100 ng/ml) or with live bacteria or heat-killed (HK) bacteria at a multiplicity of infection (MOI) of 10 (bacteria:cells) in a complete culture media for 6–24 h, as indicated. HK bacteria were obtained by heating the bacteria at 95°C for 45 min and complete killing was confirmed by culturing the lysate on chocolate agar plates. After the indicated time, macrophage culture supernatants were collected to detect the presence of various cytokines. RNA was extracted from the adherent cells by RNeasy mini spin column (Qiagen Sciences, Germantown, MD) and used for real-time RT-PCR. For experiments involving various MAP kinase inhibitors, total splenocytes were isolated from TLR4^−/−^ mice and cultured in 24 well tissue culture plate at 2×10^5^ cells/well. Prior to stimulation with live or HK bacteria, cells were pretreated with p38 MAP kinase inhibitor SB202190 (10 µM), JNK1/2 inhibitor SP600125 (10 µM) and ERK1/2 inhibitor U0126 (10 µM) for 1 h followed by treatment with live or HK bacteria. After 18 h, culture supernatants were collected and used for cytokines measurement.

For experiments involving various MAP kinase inhibitors, splenocytes were isolated from WT or TLR4^−/−^ mice and cultured in 24 well tissue culture plate at 2×10^5^ cells/well. Prior to stimulation with live or HK bacteria, cells were pretreated with p38 MAP kinase inhibitor SB202190 (10 µM), JNK1/2 inhibitor SP600125 (10 µM), or ERK1/2 inhibitor U0126 (10 µM) for 1 h followed by treatment with live or HK bacteria. After 18 h, culture supernatants were collected for cytokine measurement.

### Detection of Cytokines by ELISA

The levels of TNF-α, IL-6, IL-12 p40, IFN-β, and IFN-γ in culture supernatants in response to LOS, live or HK *M.cat* were determined using enzyme-linked immunosorbent assay (ELISA) kit (R&D Systems, Minneapolis, MN) according to the manufacturer's instruction.

### Real time RT-PCR

Total RNA extracted from previous steps were used to generate first-stand cDNA by TaqMan reverse transcriptase reagents (Applied Biosystem, Foster city, CA) using Eppendorf Mastercycler (Eppendorf, Hamburg, Germany). The reverse transcribed cDNA were amplified and quantified using power SYBR green PCR master mix by Step One plus real time PCR system (Applied Biosystem) with specific primers according to the manufacturer's protocol. The primer sequences were used as follows: RANTES, 5-CTCACCATATGGCTCGGACA-3 (Forward primer), 5-CTTCTCTGGGGCACACA-3 (Reverse Primer), Actin, 5-AGCTGCGTTTTACACCCTTT-3 (Forward primer), 5-AAGCCATGCCAATGTTGTCT-3 (Reverse Primer). Following the amplification, melting curve analysis was performed at temperature between 60°C to 95°C.

### Bacterial challenge and sampling

TLR4 mutant C3H/HeJ and control C3H/OuJ mice were challenged with aerosolized *M. catarrhalis* strain 25238 at 1×10^8^ CFU/ml in an inhalation exposure system (Glas-Col, LLC, Terre Haute, IN) as previously described [28]. At 3, 6, 12, and 24 h post-challenge, mice were euthanized and lungs were harvested and homogenized in 1 ml of phosphate buffered saline (PBS) with a Precellys 24 homogenizer (Bertin Technologies, Paris, France). Serially diluted lung homogenates were cultured on chocolate agar plates overnight at 37°C with 5% CO_2_ to determine colony forming units (CFU). The remainder of the lung homogenates were used for cytokine measurements. Six mice per strain were used at each time point.

### Statistical analysis

Statistical significance was determined by student's *t-test*. Experimental results are expressed as the mean of duplicates ± standard deviation in at least 2 independent experiments. The pulmonary levels of viable bacterial counts were logarithm transformed ((log)_10_ CFU) for statistical analysis. The difference between the lung viable bacterial counts from wild-type and that from TLR4 mice at each time point was analyzed by using Student's t-test.

## Results

### CD14-TLR4 contribute to initiate LOS-induced host responses in murine macrophage

**Figure 1:**
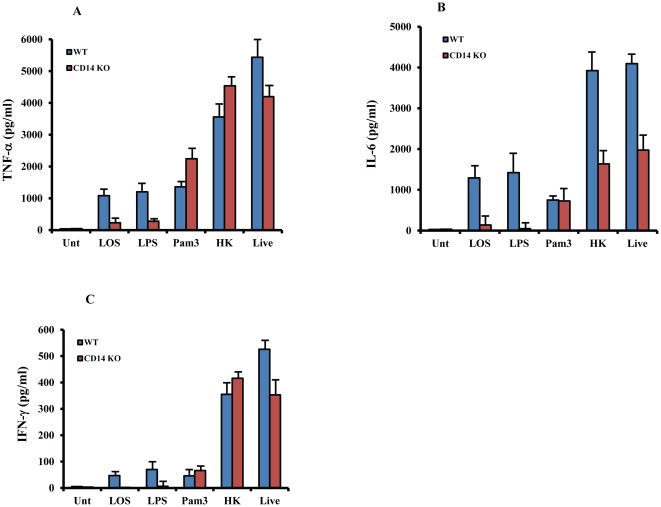
Membrane bound CD14 is required for LOS but not whole *M.cat* induced host response. (A, B) BMMØ, (C) total splenocytes were generated from CD14 knock-out and WT control mice (C57BL/6J) and cultured in 24 well tissue culture plate at 2×10^5^ cells/well as described in materials and methods. Cells were then treated with LOS (100 ng/ml), LPS (100 ng/ml), Pam3 (10 µg/ml), HK and live *M.cat* (m.o.i-1∶10) for 6 and 18 hours, respectively. Supernatants were collected and the levels of TNF-α (A), IL-6 (B) and IFN-γ (C) were measured by ELISA. Representative data from two independent experiments are shown.

CD14 initiates the LPS signaling through a physical interaction with TLR4-MD2 complex [29]. We first investigated the relative contribution of CD14 in macrophages stimulated by LOS or by HK and live *M.cat*. BMMØ generated from CD14^−/−^ and WT (C57BL/6J) mice were treated with *M. cat* LOS (100 ng/ml), *E. coli* LPS (100 ng/ml), the TLR2 ligand Pam3CSK4 (10 µg/ml), and live or HK *M.cat* for 6 hours and levels of TNF-α and IL-6 in culture supernatants were measured using ELISA. Treatment with both LPS and LOS resulted in secretion of TNF-α and IL-6 from macrophages isolated from WT mice that was significantly reduced in CD14-deficient macrophages (Fig. 1A–B). In contrast, the induction of TNF-α and IL-6 by the TLR2 ligand was completely CD14-independent yet induced roughly equivalent levels of both TNF-α and IL-6 as the LPS and LOS. However, WT and CD14^−/−^ macrophages treated with live or HK *M. cat* produced much greater levels of TNF-α, and IL-6 than the other stimuli; however, TNF-α production was CD14-independent, while IL-6 production was partially inhibited in CD14^−/−^ macrophages compared to WT macrophages. Next we treated total splenocytes from WT and CD14^−/−^ mice in a similar fashion and compared IFN-γ secretion between these 5 treatments. Similar to TNF-α and IL-6, IFN-γ was poorly inducible in either LPS- or LOS-treated WT macrophages, and not inducible in CD14-deficient macrophages. CD14-deficient macrophages treated with live or HK *M.cat* showed strong production of IFN-γ compared to WT macrophages. As seen in Fig. 1A, B, a lack of CD14 had no effect on Pam3CSK4- induced IFN-γ secretion (Fig. 1C).

**Figure 2:**
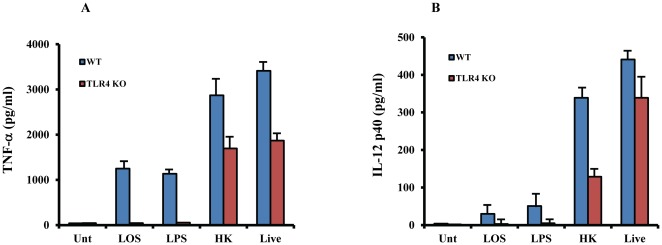
Partial contribution of TLR4 in *M. cat* induced host response. BMMØ were generated from TLR4-deficeint and WT control mice (C57BL/6J) and cultured in 24 well tissue culture plate at 2×10^5^ cells/well. Cells were then treated with LOS (100 ng/ml), LPS (100 ng/ml), HK and live *M.cat* as described for 6 h (A) or 18 h (B). Supernatants were collected and the levels of TNF-α (A) and IL-12p40 (B) were measured by ELISA. Representative data from two independent experiments are shown.

Next, we investigated relative contribution of TLR4 in LOS- or *M. cat*-induced macrophage responses. BMMØ generated from TLR4^−/−^ mice were treated as described above. Both LOS- and LPS-induced production of TNF-α was completely abolished in TLR4^−/−^ macrophages compared to WT macrophages (Fig. 2A). However, unlike CD14^−/−^ macrophages, where no reduction of TNF-α was seen in cells treated with either HK or live *M. cat*, we observed a modest reduction of TNF-α secretion in TLR4^−/−^ macrophages compared to WT control cells. To confirm that TLR4 signaling is only partially responsible for the HK- or live *M.cat*-induced host response, we measured a second cytokine, IL-12 p40, and obtained similar data (Fig. 2B). Together, these data show that the CD14-TLR4 signaling axis is absolutely required for the *M. cat* LOS-induced host response, but does not account fully for the host response to either live or HK *M. cat*.

### *M.cat* induced host response depends partially on TLR2

**Figure 3:**
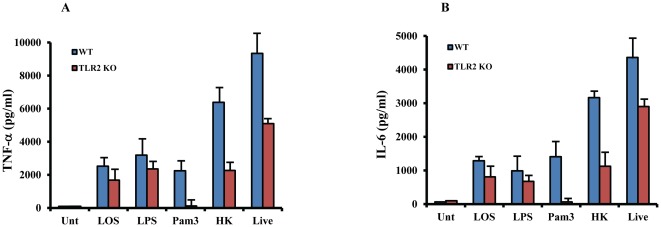
Partial contribution of TLR2 in *M. cat* induced host response. BMMØ were generated from TLR2-deficient and WT control mice (C57BL/6J) and cultured in 24 well tissue culture plate at 2×10^5^ cells/well. Cells were then treated with LOS (100 ng/ml), LPS (100 ng/ml), Pam3 (10 µg/ml), HK and live *M.cat* as described in materials and methods for 6 h. Supernatants were collected and the levels of TNF-α (A) and IL-6 (B) were measured by ELISA. Representative data from two independent experiments are shown.

Although the cell wall of *M. cat* is mostly composed of LOS, it also contains lipoproteins that may initiate cellular response through TLR2. To determine the role of TLR2 in *M.cat*-induced host responses, we generated BMMØ from TLR2^−/−^ mice and treated cells with LOS, LPS, HK and live *M.cat* for 6 hours and quantified cytokines secretion in culture supernatants. There were no significant differences observed in either LOS- or LPS-induced TNF-α or IL-6 secretion between WT and TLR2^−/−^ macrophages (Fig. 3A–B). These data confirm that TLR2 has no role in LOS-induced host response. On the other hand, similar to TLR4, we observed partial reduction of both HK- and live *M.cat-*induced TNF-α and IL-6 secretion in TLR2^−/−^ macrophages. Pam3CSK4 was used as a synthetic ligand for TLR2 and failed to induce cytokine secretion in TLR2^−/−^ macrophages compared to WT control. Together, these data show that TLR2 is partially responsible for *M. cat*-induced host responses.

### *M.cat* initiates host response through TLR2/TLR4/TLR9

**Figure 4:**
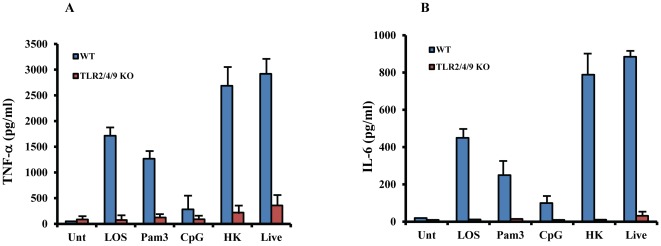
Recognition of *M.cat* by host depends on combined effect of TLR2/4/9. BMMØ were generated from TLR2/4/9 triple knock-out and WT control mice (C57BL/6J) and cultured in 24 well tissue culture plate at 2×10^5^ cells/well. Cells were then treated with LOS (100 ng/ml), Pam3 (10 µg/ml), CpG DNA (20 µg/ml), HK and live *M.cat* for 6 h. Supernatants were collected and the levels of TNF-α (A) and IL-6 (B) were measured by ELISA. Representative data from two independent experiments are shown.

Bacterial DNA often contains unmethylated CpG containing DNA motifs that can be specifically recognized by TLR9. In fact, pneumococcal and NTHi DNA have been reported as virulence factors in pathogenesis caused by *S.pneumoniae* and nontypeable *Haemophilus influenza*, respectively [14], [30]. Since we found only partial reduction of *M.cat*-induced cytokine secretion in both TLR2- and TLR4-deficient cells, we hypothesized that there must be additional receptors responsible for host response and *M. cat* DNA might be another potent virulence factor. In order to test our hypothesis, we generated TLR2^−/−^/TLR4^−/−^/TLR9^−/−^ triple knockout mice. We treated the BMMØ as described earlier with LOS, Pam3CSK4, CpG DNA, live and HK *M.cat* and measured TNF-α and IL-6 in culture supernatants after 6 hours of incubation. As expected, LOS, Pam3CSK4, and CpG DNA induced production of both TNF-α and IL-6 was completely abolished in TLR2^−/−^/TLR4^−/−^/TLR9^−/−^ triple knockout macrophages compared to WT control cells (Fig. 4 A–B). In addition, we found TLR2/4/9 triple knockout macrophages secreted minimal levels of cytokines in response to either live or HK *M. cat*. Together, these critical data confirm that host response against *M. cat* depends on combined recognition of TLR2, TLR4, and TLR9.

### *M.cat* induces host response through both MyD88- and TRIF-dependent signaling pathway

**Figure 5:**
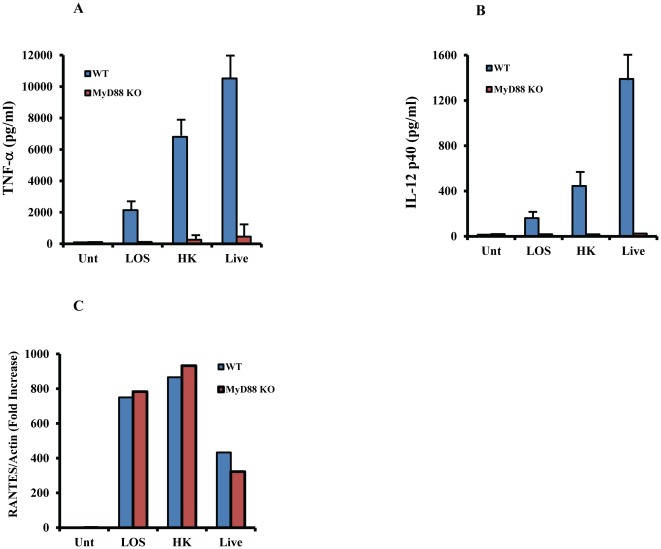
Impaired immune response to *M.cat* by MyD88 deficient macrophages. BMMØ were generated from MyD88 knock-out and wild type control mice (C57BL/6J) and cultured in 24 well tissue culture plate at 2×10^5^ cells/well. Cells were then treated with LOS (100 ng/ml), HK and live *M.cat* for 6 h (A) or 18 h (B, C). **A–B**, Supernatants were collected and the levels of TNF-α (A) and IL-12p40 (B) were measured by ELISA. **C.** Total RNA was extracted after 18 h of *M.cat* treatment and gene expression of RANTES was analyzed by real time RT-PCR. Representative data from three independent experiments are shown.

After recognition of bacterial pathogen by various TLRs, distinct intracellular pathways are activated that include different adapter molecules such as MyD88 and Toll/IL-1R domain-containing adaptor protein inducing IFN-β (TRIF) [3]. All TLRs (TLR2, TLR4, TLR9) that are responsible for *M. cat* recognition share a common adapter molecule, MyD88. We next investigated the function of MyD88 in *M. cat*-induced host responses in murine macrophages. WT and MyD88^−/−^ BMMØ were treated with LOS and live or HK *M.cat* and measured production of TNF-α and IL-12 p40 in culture supernatants. As expected, secretion of both the cytokines was severely impaired in MyD88^−/−^ mice compared to WT control cells (Fig. 5 A–B). To confirm that MyD88^−/−^ macrophages have a functional MyD88-independent signaling pathway, we measured gene expression of RANTES, a cytokine that exclusively depends on the adapter molecule TRIF. As expected, there was no difference in RANTES gene expression in both WT and MyD88^−/−^ macrophages in response to *M. cat*-derived stimulation (Fig. 5C).

**Figure 6:**
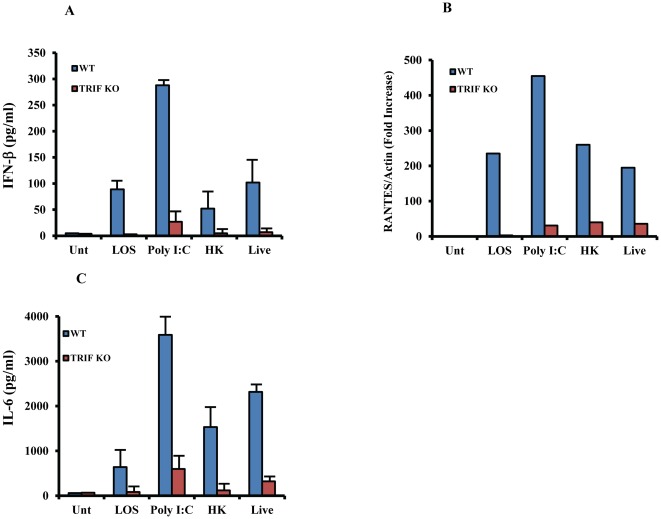
Impaired immune response to *M.cat* by TRIF-deficient macrophages. Bone marrow derived macrophages were generated from TRIF knock-out and wild type control mice (C57BL/6J) and cultured in 24 well tissue culture plate at 2×10^5^ cells/well. Cells were then treated with LOS (100 ng/ml), Poly I:C (50 µg/ml), HK and live *M.cat* for 18 h (A–B) or 6 h (C). **A, C**, Supernatants were collected and the levels of IFN-β (A) and IL-6 (C) were measured by ELISA. **B.** Total RNA was extracted after 18 h of *M.cat* treatment and gene expression of RANTES was analyzed by real time RT-PCR. Representative data from two independent experiments are shown.

Among all the TLRs, only TLR4 has the unique ability to initiate signaling pathway through both MyD88- and TRIF-dependent pathways [4]. TRIF is also the adapter utilized by TLR3 that recognizes double-stranded RNA. Since *M.cat* stimuli is recognized by TLR4 (Fig. 2), it was reasonable to hypothesize that *M.cat* also has the ability to initiate TRIF-dependent host response. In fact, Fig. 6 shows that LOS, and live and HK *M.cat* induce secretion of IFN-β and RANTES (both are TRIF-dependent) gene expression in WT macrophages. However, the levels of IFN-β and RANTES were severely impaired in macrophages generated from TRIF^−/−^ mice. The TLR3 ligand Poly I:C was used as a positive control for this study. Interestingly, we found a complete reduction of IL-6 secretion in response to *M.cat* stimuli in TRIF^−/−^ macrophages compared to WT cells (Fig. 6C). An earlier study by Yamamoto *et al.* also showed that inflammatory cytokines, including IL-6, was severely impaired in TRIF-deficient macrophages in response to TLR4 ligand, but not to other TLR ligands [6].

### Inhibition of MAP kinase impairs *M.cat* induced cytokine secretion

**Figure 7:**
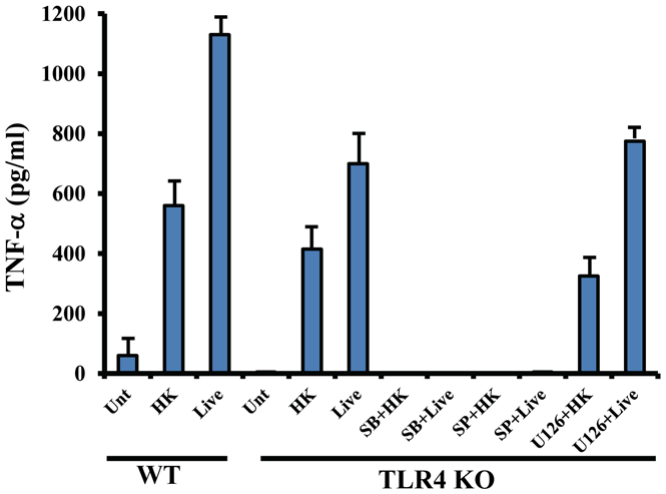
*M.cat* induces host response through TLR4-MAP kinase signaling pathway. Total splenocytes were isolated from TLR4 knock-out and WT control mice (C57BL/6J) and cultured in 24 well tissue culture plate at 2×10^5^ cells/well and pretreated with p38 inhibitor SB202190 (10 µM), JNK1/2 inhibitor SP600125 (10 µM) and ERK1/2 inhibitor U0126 (10 µM) for 1 h followed by treatment with either HK or live *M.cat* for 18 h. Supernatants were collected and the levels of TNF-α were measured by ELISA. Representative data from two independent experiments are shown.

Once TLRs are activated, they recruit the MyD88 adapter protein that, in turn, recruits the IL-1 receptor to IR-1R-associated protein kinase (IRAK-1). IRAK-1 becomes phosphorylated and subsequently degraded leading to recruitment of TRAF6 to the growing receptor complex. This process results in the activation of mitogen activated protein kinase (MAPK) that involve the c-Jun NH_2_-terminal (JNK1/2) kinase, extracellular signal-regulated kinase (ERK1/2) and p38 mitogen activated protein (MAP) kinase and transcription factor NF-κB [31]. Previously, we found that TLR2 and TLR4 are only partially responsible for *M.cat*-induced TNF-α secretion (Fig. 2, 3). Based on the above data, we hypothesized that *M.cat* most likely activates MAPK, in addition to recognition by TLR4. To test our hypothesis, we took total splenocytes from TLR4-deficient mice and pretreated them with various MAP kinase inhibitors such as SP600125 (JNK1/2 kinase inhibitor), SB202190 (p38 MAP kinase inhibitor), and U0126 (ERK1/2 kinase inhibitor), followed by stimulation with HK and live *M.cat*, and measured TNF-α secretion in the culture supernatants. Similar to BMMØ (Fig. 2), total splenocytes from TLR4-deficient macrophages showed partial reduction of TNF-α secretion compared to WT control macrophages (Fig. 7). However, in TLR4-deficient cells pretreated with either JNK1/2 or p38 inhibitor, absolutely no TNF-α was found in culture supernatant. In contrast, inhibition of ERK1/2 had no effect on *M.cat*-induced TNF-α production. Together, these data showed that *M.cat* is able to initiate host response through TLR4-MAPK signaling pathway in murine macrophages.

### TLR4 contributes to pulmonary clearance of *M.cat in vivo*

**Figure 8:**
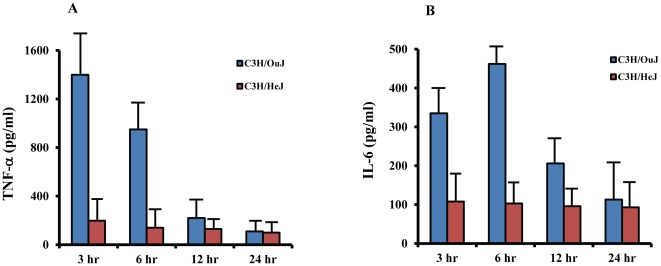
Reduced cytokines production in lung from TLR4 mutant mice in response to live *M.cat in vivo*. TLR4 mutant mice C3H/HeJ and wild type control mice C3H/OuJ were challenged with live *M.cat* type A strain at 1×10^8^ CFU/ml in an aerosol chamber as described in materials and methods. Lungs were collected at 3, 6, 12 and 24 h, homogenized and levels of TNF-α (A) and IL-6 (B) were measured by ELISA. (n = 6 for each time point).

Having established that *M.cat* initiates host inflammatory response through multiple toll like receptors and TLR4 at least partially responsible for the production of pro-inflammatory cytokines, we were interested to study the relative contribution of TLR4 in host defense against *M.cat in vivo*. TLR4 mutant mice C3H/HeJ and control mice C3H/OuJ were challenged with *M. catarrhalis* type A strain in an aerosol chamber and then the mice were euthanized at 3, 6, 12, and 24 h of post challenge, lungs were collected, homogenized and used for cytokine detection and also cultured on chocolate agar plates for bacterial counts. Live *M.cat* induced high levels of TNF-α in lung homogenates from WT mice as early as 3 h of post-challenge and then the level of TNF-α gradually decreased (Fig. 8A). Within 12 h, very little TNF-α was found and was almost absent at 24 h of post-challenge. On the other hand, secretion of TNF-α in response to *M.cat* was severely impaired in lungs from TLR4 mutant, C3H/HeJ mice. In fact, *M.cat* induced TNF-α was more significantly reduced *in vivo* compared to *in vitro*. Levels of IL-6 production in lung homogenate were also examined. Fig. 8B shows that *M.cat* induced production of IL-6 in WT mice as early as 3 h, but reached its peak at 6 h post-challenge then slowly decreased, but remained well above background levels, even after 24 h. IL-6 production was also significantly reduced in lungs from TLR4 mutant mice compared to WT mice (Fig. 8B). The relative contribution of TLR4 in bacterial clearance from lung was also assessed and a different trend was observed between WT and TLR4-defective mice. At earlier time points, *M.cat* was cleared more efficiently and significantly in TLR4 mutant mice compared to wild type mice and this trend continued until 6 h (*p<0.05*). However, at 12 h, bacterial loads were two times higher in mutant mice than in wild type control mice and remains significantly high at 12 h (*p<0.001*) and even after 24 h of post-challenge (Table 1). Together, these data provide strong evidence that multiple TLRs play a key role in *M.cat* induced host inflammatory response and mutation of TLR4 gene impairs the host ability to efficiently clear the bacterial loads from lungs *in vivo*.

**Table 1:** Pulmonary clearance patterns in TLR4 mutant C3H/HeJ and wild type control C3H/OuJ mice after an aerosol challenge with *M. catarrhalis* type A.

Time	WT	TLR4 mutant
3 h	47500±5708	34333±2124^ns^
6 h	29666±3018	15166±1518*
12 h	1975±386	3950±423**
24 h	42±5	204±65**

*Post-challenge* a *bacterial Recovery* b *[CFU/lung (SD range)].*

a Groups of six mice at each time point were challenged with 1×10^8^ CFU of *M.cat* in an aerosol chamber.

b Number of viable bacteria that are recovered at 3, 6, 12 and 24 hours.

* Significant decrease of number of bacteria was observed in TLR4 mutant mice (*p<0.05 vs* WT control).

** Significant increase of number of bacteria was observed in TLR4 mutant mice (*p<0.001 vs* WT control).

ns Not significant.

Data are expressed as mean ±SEM.

## Discussion

*Moraxella catarrhalis* is an unencapsulated, Gram-negative human respiratory pathogen. There are total of three serotypes of *M.cat*, with type A being most prevalent (61%), followed by type B and C (29% and 5%) [32]. In the present study, purified LOS from *M.cat* type A (ATCC 25238), or live or HK *bacteria* were used to stimulate murine macrophages generated from series of TLR and adapter knock-out mice and the relative contribution of each to the host response was investigated . We found that LOS and whole bacteria initiate distinct signaling pathways through one or more TLRs and is able to activate both MyD88- and TRIF-dependent signaling pathways *in vitro*. While LOS required only CD14-TLR4 complex and was able to initiate both MyD88- and TRIF-dependent host responses, both live and HK *M.cat* required multiple TLRs (TLR2, TLR4 and TLR9) and recognition of whole bacteria did not necessarily require CD14. Finally, we showed that TLR4 mutant mice produced significantly reduced levels of pro-inflammatory cytokines such as TNF-α and IL-6 in response to live *M.cat* and had significantly higher level of bacterial loads in the lungs compared to WT control mice.

Similar to *Neisseria* spp., *M. cat* possesses an LOS that is similar in structure to, but distinct from the structure of LPS that is found in the outer membrane of most Gram negative bacteria. The LOS of *M. cat* is highly conserved among 95% of known *M. cat* strains and clinical isolates [32], [33]. Despite these structural differences between LOS and LPS, we found that both induce essentially the same levels of cytokines in WT murine macrophages, CD14^−/−^ macrophages, and in TLR4^−/−^ macrophages, indicating that both the *E. coli* LPS and the *M. cat* LOS are strictly TLR4 agonists. Since LOS lacks a repeating O antigen polysaccharide, and possesses a 2 heptose “core,” its structure resembles that of a rough LPS [32]. In contrast to a previous paper claiming that smooth, but not rough, LPS preparations utilized CD14 [34], we found that CD14 is absolutely required for LOS-induced secretion of pro-inflammatory cytokines. Production of TNF-α, IL-6, and IFN-γ were severely impaired in CD14-deficient murine macrophages compared to WT cells. In contrast, CD14 was not necessary for the induction of TNF-α and IFN-γ by HK and live *M. cat* and only partially required for IL-6 production. Similar types of results also have been reported. Catharina *et al.* found elevated levels of IL-1β, IL-6, and TNF-α in lungs from CD14 knock-out mice compared to WT mice, when challenged with live NTHi [17]. However, the discrepancy between the production of TNF-α and IL-6 by *M. cat* and the role of CD14 remains elusive and needs further investigation. Once CD14 brings monomeric LPS to TLR4-MD2 complex, it initiates TLR4-mediated signaling and subsequent cytokine production. Similar to CD14, TLR4 was also found to be absolutely required for LOS-induced secretion of TNF-α and IL-12, whereas TLR4 was only partially responsible for either HK- or live *M.cat*-induced cytokine secretion, suggesting that CD14-TLR4 complex is absolutely important for host recognition of *M. cat* LOS by host, while HK or live *M. cat* are recognized by other receptors as well. Similar partial inhibition of cytokine secretion was also observed in TLR2-deficient macrophages. These results led us into search for additional TLRs that might be responsible for *M.cat* recognition. In TLR2/TLR4/TLR9 triple knockout macrophages generated from triple knockout mice failed to secrete significant levels of pro-inflammatory cytokines in response to LOS, HK, or live *M.cat* (Fig. 4). Together, these data firmly established our hypothesis that *M.cat* is recognized by host innate immunity that primarily involves TLR2, TLR4 and TLR9.

Once innate immunity is activated by various microbial stimuli through multiple TLRs, different adapter molecules bind to the intracellular domain of their specific TLR and initiate a series of intracellular signaling cascades such as MAPK and transcription factor NF-κB. MAPKs are Ser/Thr kinases that convert extracellular stimuli to intracellular signaling and regulate wide range of cellular functions such as gene expression, mitosis, metabolism, motility, survival, apoptosis and differentiation [35]. Although there are total of 14 MAPKs so far have been identified, three are most widely studied (i) extracellular signal regulated kinase 1/2 (ERK1/2), (ii) p38 MAP kinase and (iii) c-Jun N-terminal kinase 1/2 (JNK1/2). In our study, we found that prior inhibition of p38 MAPK and JNK1/2 by pharmacological inhibitor completely abolished secretion of TNF-α in response to *M.cat* stimuli in TLR4-deficient total splenocytes which otherwise was seen only partial inhibition in the same knockout mice. So far, we have provided strong evidence to establish that the host recognizes *M.cat* stimuli through CD14-TLRs as well as p38-JNK1/2. The relative contribution of two major adapter molecules, MyD88 and TRIF, in *M.cat*-induced host immune responses were also investigated. From our results, it was obvious that *M.cat* would certainly initiate the signaling pathway which would be largely dependent on MyD88 adapter molecule and found that *M.cat* induced secretion of MyD88-dependent pro-inflammatory cytokines was totally absent in macrophages generated from MyD88 deficient mice compared to control macrophages (Fig. 5). Considering the fact that LOS and LPS have different chemical structures, we were further interested to find out whether or not, LOS or HK and live *M.cat* are able to initiate MyD88-independent, TRIF dependent signaling pathway in murine macrophages. In fact, Fig. 7 shows that LOS or HK and Live *M.cat* also have the ability to induce IFN-β secretion and RANTES gene expression, two cytokines that are well known as a result of activation of TRIF dependent signaling pathway. Further, we found that *M. cat*-induced production of IFN-β and expression of RANTES was completely impaired in macrophages from TRIF-deficient mice.

Many recent studies have investigated the role of TLRs in host defense against Gram negative bacterial infection in lung. It has been found that TLR4 significantly contributes to protective immune responses [36]. Moreover, a significantly higher incidence of bacterial infection has been reported in intensive care unit patients with TLR4 mutations [37]. When NTHi was injected in the middle ear of TLR4 mutant mice C3H/HeJ, the severity of acute otitis media was prolonged to 48 hours compared to control mice [38]. Consistent with these observations, we also found that at initial time points after bacterial challenge, *e.g*., 6 hours, bacterial loads were, in fact, significantly lower in TLR4 mutant mice compared to wild type mice (*p<0.05*), despite the fact that at the same time mutant mice produced significantly less amount of pro-inflammatory cytokines. However, with time, mutant mice were clearly less able to clear *M.cat* from their lungs and at 24 hours, the number of viable bacteria was five times higher than the WT mice (*p<0.001*). It is possible that either less polymorphonuclear cells (PMNs) are recruited to lungs in mutant mice or have the impaired ability to phagocytosis the bacteria, resulting in an increased number of bacteria at the later time points. However, it is also possible that other factors such as complements play a crucial role in early reduction of bacterial loads.

In this report, we have clearly identified the specific signaling pathways that lead to activation of host immune responses in response to *M.cat* LOS or whole bacteria. We have shown that while LOS absolutely requires CD14 and TLR4, it does not require TLR2; however, whole *M. cat* shows a partial requirement for CD14, TLR2, TLR4, and TLR9, possibly reflecting the concurrent expression of distinct PAMPs on the organism. Despite the structural differences between LPS and LOS, like LPS, LOS also has the ability to induce both MyD88 dependent and TRIF dependent signaling pathway through TLR4. On the other hand, recognition of whole *M.cat* requires multiple TLRs and in addition to MyD88 and TRIF adaptor molecules, it also induces secretion of pro-inflammatory cytokines through MAP kinase.
